# Energy Metabolite, Immunity, Antioxidant Capacity, and Rumen Microbiota Differences Between Ewes in Late Gestation Carrying Single, Twin, and Triplet Fetuses

**DOI:** 10.3390/ani14223326

**Published:** 2024-11-19

**Authors:** Jiaxin Chen, Chunhui Duan, Sicong Yue, Xiaona Liu, Jinhui Li, Yingjie Zhang, Yueqin Liu

**Affiliations:** College of Animal Science and Technology, Hebei Agricultural University, Baoding 071000, China; chenjiaxin1226@163.com (J.C.); duanchh211@126.com (C.D.); yueyueyuexiaojuan@163.com (S.Y.); liuxiaonahau@163.com (X.L.); lijinhui80232020@163.com (J.L.)

**Keywords:** multi-fetal ewes, late gestation, antioxidant capacity, inflammation, rumen microbiota

## Abstract

The objective of this study was to investigate the differences in the energy metabolites, immunity, antioxidant capacity, and rumen microbiota of ewes with different numbers of fetuses. Thirty healthy ewes were selected and divided into single- (SL, *n* = 10), twin- (TL, *n* = 10), and triplet-fetal (PL, *n* = 10) ewes according to the number of fetuses. Sampling was carried out on days 21 (Q21) and 7 (Q7) before lambing. The results show no differences (*p* > 0.05) in the DMI and BW of ewes with different numbers of fetuses, and the body condition score (BCS) of PL ewes was lower (*p* < 0.05) than that of SL ewes. The concentrations of β-hydroxybutyric acid (BHBA), non-esterified fatty acids (NEFA), interleukin-2 (IL-2), interleukin-6 (IL-6), and tumor necrosis factor α (TNF-α) in the PL ewes were higher (*p* < 0.05), while the glucose (Glu), triglyceride (TG), total cholesterol (TC), superoxide dismutase (SOD), glutathione peroxidase (GSH-Px), and total antioxidant capacity (T-AOC) values were lower (*p* < 0.05) than those of the SL ewes. ANOSIM analysis showed that the rumen bacterial structure of the SL, TL, and PL ewes was different on days Q21 and Q7. The relative abundance of Firmicutes and Bacteroidota in the rumen was affected (*p* < 0.05) by the number of fetuses: the relative abundance of Firmicutes (*Ruminococcus*, *Butyrivibrio*, *Christensenellaceae_R-7_group*, *Lachnospiraceae_AC2044_group*, *Lachnospiraceae_XPB1014_group*, and *Anaeroplasma*) was higher (*p* < 0.05), while that of Bacteroidota (*Prevotella*, *Prevotellaceae_UCG-003*, and *Prevotellaceae_UCG-001*) was lower (*p* < 0.05) in the SL ewes than in the PL ewes. In summary, the rumen microbial structure and energy metabolites of ewes in late gestation with different numbers of fetuses were different. Triplet-fetal ewes were characterized by lower BCS and antioxidant capacity and were prone to the triggering of inflammatory responses.

## Simple Summary

Multi-fetal ewes are more likely to suffer from metabolic disorders and pregnancy toxemia than single-fetal ewes in late gestation. The differences in the energy metabolites, immunity, antioxidant capacity, and rumen microbiota of ewes with different numbers of fetuses are unclear. This study found that triplet-fetal ewes were characterized by a lower BCS and antioxidant capacity, and were prone to the triggering of inflammatory responses. With the increase in fetal number, the concentration of BHBA increased, and that of glucose decreased; the relative abundance of Firmicutes was lower, while that of Bacteroidota was higher in triplet-fetal ewes. The differences in the rumen microbiota may be due to differences in the utilization of feed materials by ewes with different numbers of fetuses; multi-fetal ewes tend to ingest more grain-based feed to meet their energy requirements. Therefore, special nutritional strategies and refined feeding management approaches should be developed to meet the physiological requirements of multi-fetal ewes.

## 1. Introduction

The perinatal period is characterized by hormonal, physiological, psychological, and nutritional changes in ruminants. The complex physiological adaptation process increases metabolic disorder and disease susceptibility [1], especially in late gestation. Ewes are prone to suffering from many metabolic and inflammation-related diseases, such as pregnancy toxemia [2], mastitis [3], and endometritis [4]. However, morbidity differs among individual ewes, which may be related to energy metabolism, parity, and the number of fetuses. Thus, a comprehensive understanding of the physiological processes in ewes in late gestation can reduce morbidity and mortality. Pregnancy toxemia often occurs in late gestation (2–3 weeks before lambing) [5], with a higher incidence in ewes carrying two or more fetuses [6]. The health status of ewes can be assessed through physical examinations and the measurement of the body condition score (BCS); monitoring and maintaining the BCS is an effective strategy to prevent the development of pregnancy toxemia [7].

Energy requirements increase with the increase in the number of fetuses carried by ewes during late gestation [8]. However, the rapid expansion of the uterus due to fetal growth and development leads to reduced dry matter intake (DMI) [9]. A negative energy balance (NEB) in late gestation is a risk factor for disease occurrence [10], and this process generates reactive oxygen species (ROS) that cause membrane phospholipid peroxidation to exert its cytotoxicity [11]. Therefore, detecting the status of the antioxidant defense system in ewes is a key indicator used to prevent the occurrence of disease [12,13]. A previous study found that oxidative stress occurs during lactation when the homeostasis process changes in dairy goats [14]. In addition, carrying multiple fetuses stimulates the hypothalamic–pituitary–gonadal axis to secrete more intense hormones, which may interfere with the maintenance of physiological balance and the integrity of immune function in ewes [15]. Immunosuppression in ewes can be measured in terms of cytokine release, which can promote acute-phase protein (APP) synthesis and white blood cell accumulation at inflamed sites [16]. A study found that APP and cortisol may differ between single- and multi-fetal females [17]. At the same time, the physiological adaptation process of ewes is related to fluctuations in serum metabolites, and metabolic profiling is a commonly used tool to monitor metabolic health status [18]. Cabiddu et al. found that multiparous ewes have a larger litter size and lower concentrations of glucose (Glu), cholesterol (TC), and triglycerides (TG). Moreover, the parameters of inflammation and oxidative stress differ between primiparous and multiparous ewes [19]. Other studies have reported changes in the concentration of energy metabolites in ewes with different fetal numbers, mainly used to assess the relationship between metabolic status and disease in ewes [20,21,22].

A previous study found a correlation between serum metabolite changes and rumen microbiota [23]. Moreover, the gastrointestinal microbiota is critical for epithelial cell function and antioxidant and cytokine production [24]. The immune and antioxidant capacity of dairy cows can be improved by regulating the rumen microbiota [25]. Pregnancy toxemia can change the structure of the rumen microbiota in ewes [2], indicating that the rumen microbiota may reflect the metabolic and health status of ewes. In addition, probiotics can improve the reproductive performance of sows and laying hens through the gastrointestinal microbiota [26,27]. The maternal microbiota can regulate the development of the placenta and affect fetal growth [28], and there is a strong correlation between the litter size of sows and the intestinal microbiota [29]. Although previous studies indicated that the gastrointestinal microbiota changes during the perinatal period in ewes [30,31,32], it is unclear whether there are differences in the rumen microbiota of ewes with different numbers of fetuses.

As the number of fetuses increases, the metabolic requirements for the maternal and fetus also increase, raising the risk of metabolic diseases and affecting the ewe’s immune and oxidative status. Metabolic disorders in ewes generally occur in the late gestation period. Therefore, we hypothesized that the serum parameters and rumen microbiota of ewes undergo drastic changes during this period. In order to better understand the physiological changes in ewes with different numbers of fetuses, in this study, we analyzed differences in the energy metabolites, immune and oxidation status, and rumen microbiota of ewes carrying different numbers of fetuses in late gestation and provide basic information on nutrient regulation and metabolic disease prevention in multi-fetal ewes.

## 2. Materials and Methods

### 2.1. Animals and Management

The Ethics Committee of Hebei Agricultural University approved all procedures involving animals in this study (License No. 2024360). The animal experiment was carried out over a 21-day period (September 2021) at Lanhai Animal Husbandry Technology Co., Ltd., in Zhangjiakou, Hebei, China (42°62′25″ N, 115°16′ 50″ E). The temperature was maintained at 23–25 °C throughout the study period, and the mean relative humidity was maintained at 55%. The ewes were examined using standard clinical procedures [33] to exclude those with clinical symptoms and history prior to pregnancy. One hundred ewes were inserted with a progesterone plug (MAP, 45 mg/sheep, SYNCRITE-45 Vaginal Sponge, Ascot Vale, VIC, Australia) for 12 days. The injection of equine chorionic gonadin (eCG) induced estrus, and the same ram (Hu sheep) was selected for artificial insemination (AI). Ultrasound examination procedures were used to determine whether the ewes were pregnant. Fifty healthy ewes (Hu sheep, 120 days of gestation) at second parity were fed separately, with each ewe in a single barn (2.0 × 1.3 m^2^), and each ewe was considered an independent experimental unit. Since the ovulation time of the ewes was synchronized, AI was arranged according to the expected ovulation time; therefore, the lambing time for all ewes was similar. The number of fetuses were determined on day 120 of gestation via transabdominal ultrasonography (HS-1600V-7.5 MHz, Aichi, Japan), which was verified after the ewes lambed. The gestation period of each ewe was calculated from mating and lambing records (SL, 146.29 ± 1.70; TL, 145 ± 1.90; PL, 146.17 ± 1.69). Finally, single (SL, *n* = 10), twin (TL, *n* = 10), and triplet (PL, *n* = 10)-fetal ewes were selected to study the differences in energy metabolites, immune and oxidation status, and rumen microbiota on days 21 (Q21) and 7 (Q7) before lambing. The experimental ewes were maintained in a single sheepfold with the same management conditions, had ad libitum access to fresh water, and were fed twice daily (8:00 and 17:00) with a total mixed ration, as shown in Table 1. The ewes were fed libitum for 5 days (from day 120 to 125 of gestation) to determine the feed intake baseline, ensuring that rejected feed accounted for 3–5% of the provided feed.

### 2.2. Sample Collection and Chemical Analysis

Feed samples were collected weekly and stored at −20 °C for further moisture and chemical composition analysis. The DMI of the ewes was recorded daily from day 125 of gestation until lambing, as determined by the difference between the feed provided and rejected. Blood and rumen fluid were collected, and body weight (BW) measurements were performed at 07:00 am (i.e., before feeding) on days Q21 and Q7. The ewe BCS was assessed as described by Jefferies by a single operator at each research site [34], using 0.5 score increments.

The feed samples were ground, weighed, and passed through a 40-mesh sieve and dried at 105 °C for 3 h. The DM (method 930.15), crude protein (CP, method 968.06), ether extract (EE, method 973.18), calcium (Ca, method 935.13) and phosphorus (P, method 965.17) were determined according to the methods of the Association of Official Analytical Chemists (AOAC) [35]. Concentrations of neutral detergent fiber (NDF) and acid detergent fiber (ADF) were determined using filter bags and fiber analysis equipment (Ankom A200; Ankom Technology, Macedon, NY, USA) based on the method described by Van Soest et al. [36], while the NDF was evaluated using heat-stabilized alpha-amylase. Rumen fluid samples were collected from ewes using an oral cannula (Kelibo Animal Husbandry Technology Co., Ltd., Wuhan, China). The oral cannula was thoroughly cleaned with fresh warm water between sample collections, and the first 20 mL of rumen fluid was discarded to avoid saliva contamination. The collected fresh rumen fluid samples were put into 2 mL sterile tubes, quickly frozen in liquid nitrogen, transferred to the laboratory, and stored at −80 °C until DNA extraction. Blood samples (10 mL) were collected from the jugular vein and stored in a vacuum tube. The samples were centrifuged at 3000× *g* for 15 min at 4 °C to obtain serum and stored at −20 °C. Serum glucose (Glu), β-hydroxybutyric acid (BHBA), non-esterified fatty acids (NEFA), triglycerides (TG) and total cholesterol (TC), activities of superoxide dismutase (SOD), total antioxidant capacity (T-AOC), and glutathione peroxidase (GSH-Px) in the serum were measured using commercial colorimetric assay kits (Kaminuo Biology Co., Nanjing, China) following the procedure suggested by the manufacturer. The serum malondialdehyde (MDA) concentration was measured via the thiobarbituric acid method using a commercial kit (Kaminuo Biology Co., Nanjing, China). Cytokines (interleukin-1β (IL-1β), interleukin-2 (IL-2), interleukin-6 (IL-6), and tumor necrosis factor α (TNF-α)) were determined with ELISA test kits (Kaminuo Biology Co., Nanjing, China) according to the manufacturer’s instructions.

### 2.3. DNA Extraction and Sequencing

A total of 2 mL rumen fluid from each sample was used for genomic DNA extraction. The DNA was isolated and purified using an OMEGA Stool DNA Kit (Omega Bio-tek, Inc., Norcross, GA, USA) following the procedure suggested by the manufacturer. A Nanodrop 2000 spectrophotometer (Thermo Scientific, Wilmington, DE, USA) and 1% agarose gel electrophoresis were used to check the DNA concentration and quality. The absorption ratio (OD_260_/OD_280_) of the genomic DNA was greater than 1.80, indicating high DNA purity, and the DNA sample concentration was adjusted to 20 ng/µL. To construct the 16S amplicon libraries, the V3–V4 hypervariable region of the bacterial 16S rDNA was characterized using 341F (5′-CCTAYGGGRBGCASCAG-3′) and modified 806R (5′-GGACTACNNGGGTATCTAAT-3′) primers [37]. A mastermix for amplification was prepared using KAPA 2G Robust Hot Start Ready Mix and 25 µL reaction volumes. Then, 5 µL DNA (total template quantity of 30 ng), 1 µL of each primer (5 µM), and 5.5 µL H_2_O were added. The thermocycling protocol of the amplification was as follows: denaturation at 95 °C for 5 min, followed by 32 cycles of 95 °C for 45 s, annealing at 55 °C for 50 s, and elongation at 72 °C for 45 s with a final extension at 72 °C for 10 min. The expected size of the PCR products was determined using 1% agarose gel electrophoresis. The amplified PCR products were purified using an Agencourt AMPure XP Kit (Beckman Coulter Genomics, Indianapolis, IN, USA) and quantified using PCR (ABI 9700, Thermo Fisher Scientific, Waltham, MA, USA). Purified PCR products were pooled in equimolar amounts and sequenced on an Illumina MiSeq (Illumina, San Diego, CA, USA) paired-end 300 sequencing platform at Allwegene Company (Beijing, China).

### 2.4. Bioinformatics Analysis

To obtain accurate and reliable results, the Quantitative Insights into Microbial Ecology (QIIME) procedure (v1.9.1) was used to eliminate low-quality sequences (quality score < 20 and length < 225 bp). At the same time, Pear (V0.9.6) software was used to filter and splice the data; the minimum overlap was set to 10 bp, and the mismatch rate was 0.1. The data were then clustered into operational taxonomic units (OTUs) with 97% similarity, and the chimeras and error sequences were removed from the optimized data. The observed OTUs were calculated using the Shannon index, the α diversity was evaluated via phyloseq using the Chao1 index, and sparse curves were drawn using R (v3.0.3) software. Principal component analysis (PCoA) was performed on a Bray–Curtis distance matrix using the ANOSIM method in QIIME.

### 2.5. Statistical Analyses

The different time points, Q21 and Q7 (21 and 7 days before lambing), were considered as different time treatments according to the Repeated Measurement Model, and the sampling time was calculated according to the lambing date of the ewes. SPSS 22.0 (SPSS Inc., Chicago, IL, USA) was used to test the independence, normality, and uniformity of the DMI, BW, BCS, serum parameters, α-diversity, and rumen bacteria, then the evaluations were processed using one-way and two-way ANOVA. The two-way ANOVA was used to evaluate the sampling time and interactions between groups, and the analytical model was as follows: Yij = μ + Xi + Zj + XZij + eij, where Yij is the dependent parameter, μ is the overall mean, Xi is the fixed effect of group (SL, TL, or PL), Zj is the fixed effect of time (day Q21 or Q7) before lambing, XZij is their interaction, and eij is the residual error. When significant effects between groups were detected, the means were compared using the LSD test, and significant effects were declared at *p* < 0.05.

## 3. Results

### 3.1. Differences in the DMI, BW, and BCS of Ewes in Late Gestation with Different Numbers of Fetuses

The DMI and BW of the ewes in late gestation with different numbers of fetuses are shown in Table 2. No differences (*p* > 0.05) were found in the DMI and BW of the ewes. However, the number of fetuses and gestation time had significant effects on the BCS of the ewes, but no interactions (*p* > 0.05) between these two factors were observed, and the BCS of the PL ewes was lower (*p* < 0.05) than that of the SL ewes in late gestation.

### 3.2. Differences in the Energy Metabolites of Ewes in Late Gestation with Different Numbers of Fetuses

The serum energy metabolite parameters of ewes in late gestation with different numbers of fetuses are shown in Table 3. These parameters were not affected (*p* > 0.05) by the interaction between the number of fetuses and gestation time, but BHBA, NEFA, Glu, TG, and TC were affected (*p* < 0.05) by the number of fetuses. The concentrations of BHBA and NEFA in the PL ewes were higher (*p* < 0.05) than those of the SL ewes, and the concentration of Glu increased (*p* < 0.05) along with the increase in the number of fetuses. The concentrations of TG and TC in the PL ewes were lower (*p* < 0.05) than those of the SL ewes.

### 3.3. Differences in the Serum Immune Cytokines of Ewes in Late Gestation with Different Numbers of Fetuses

The serum immune cytokine levels of ewes in late gestation with different numbers of fetuses are shown in Table 4. The concentrations of IL-2, IL-6, and TNF-α were affected (*p* < 0.05) by the number of fetuses, and these concentrations were higher in the PL ewes (*p* < 0.05) than in the SL ewes. The immune cytokines were affected (*p* < 0.05) by gestation time: the concentrations of IL-1β, IL-2, IL-6, and TNF-α were higher (*p* < 0.05) on day Q7 than that on day Q21. No interactions (*p* > 0.05) were observed between the number of fetuses and time of gestation.

### 3.4. Differences in the Serum Antioxidant Indices of Ewes in Late Gestation with Different Numbers of Fetuses

The serum antioxidant indices of ewes in late gestation with different numbers of fetuses are shown in Table 5. The MDA was significantly affected (*p* < 0.05) by both the number of fetuses and gestation time, but no interactions (*p* > 0.05) between these two factors were observed. The concentration of MDA in the PL ewes was higher (*p* < 0.05) than that in the SL ewes. The concentrations of T-AOC, SOD, and GSH-Px were affected by the number of fetuses, and they were lower (*p* < 0.05) in the PL ewes than in the SL ewes.

### 3.5. Summary of the Rumen Communities of Ewes in Late Gestation with Different Numbers of Fetuses

**Figure 1:**
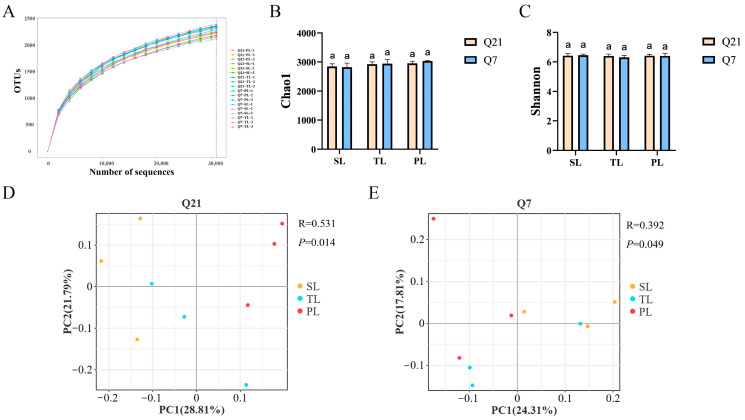
Rumen microbiota of ewes in late gestation with different numbers of fetuses. (**A**) The rarefaction curve of ewes with different numbers of fetuses in late gestation. (**B**) Chao1 index and Shannon index (**C**) for the rumen. Principal coordinate analysis (PCoA) of rumen microbiota from ewes with different numbers of fetuses on days Q21 (**D**) and Q7 (**E**).

**Figure 2:**
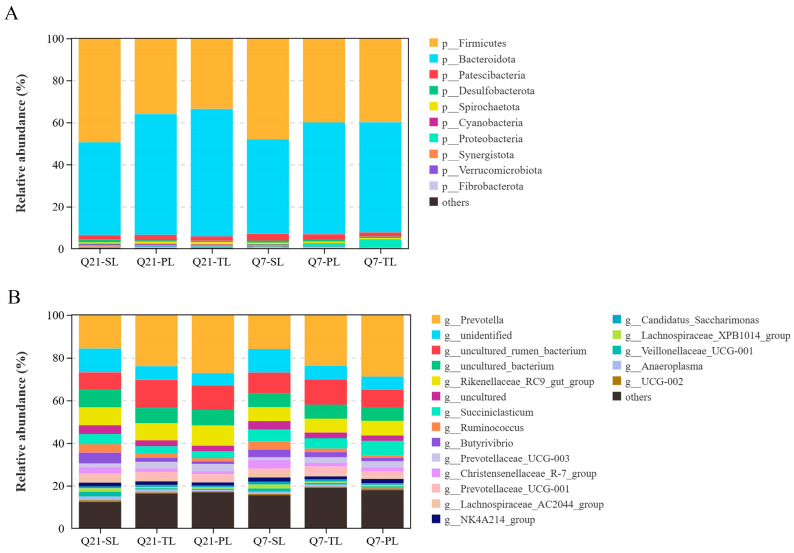
Stacked bar graphs of relative abundance of rumen microbiota at the phylum and genus levels from ewes in late gestation with different numbers of fetuses. (**A**) Stacked bar graphs of the average relative abundances of the phyla of ewes in late gestation with different numbers of fetuses. (**B**) Stacked bar graphs of the relative abundances of genera of ewes in late gestation with different numbers of fetuses.

In order to explore the differences in the rumen microbiota of ewes in late gestation with different numbers of fetuses, the study conducted high-throughput sequencing of the rumen fluid samples obtained from ewes on days Q21 and Q7. The rarefaction curves for the ewes in the three groups tended to be stable in the two periods (Figure 1A), indicating that the number of sequenced samples was reasonable enough to reflect the structure and number of rumen bacterial communities in the SL, TL, and PL ewes. The Shannon (Figure 1B) and Chao1 (Figure 1C) indices for the ewes in the three groups showed no difference (*p* > 0.05) in late gestation. Based on the Bray–Curtis distance, principal coordinate analysis (PCoA) was used. The ANOSIM results showed that the rumen bacterial structure of the SL, TL, and PL ewes differed on days Q21 (*p* = 0.014, R = 0.531) and Q7 (*p* = 0.049, R = 0.392). We found that Firmicutes, Bacteroidota, and Patescibacteria were the top three bacterial phyla (Figure 2A), while *Prevotella*, *unidentified*, *uncultured_rumen_bacterium*, *uncultured_bacterium*, and *Rikenellaceae_RC9_gut_group* were the top five bacterial genera (Figure 2B).

### 3.6. Differences in the Taxonomic Composition of Rumen Bacterial Communities of Ewes with Different Numbers of Fetuses

Focusing on dominant bacteria with a relative abundance >1%, three bacterial phyla (Table 6) and eleven bacterial genera (Table 7) in the rumen were observed to have significant differences among the ewes with different numbers of fetuses. The relative abundance of Firmicutes and Bacteroidota was affected (*p* < 0.05) by the number of fetuses, but no interaction (*p* > 0.05) was observed between the number of fetuses and gestation time. Compared with the PL ewes, the relative abundance of Firmicutes in the rumen of the SL ewes was increased (*p* < 0.05), while Bacteroidota was decreased (*p* < 0.05). The eleven dominant genera were affected (*p* < 0.05) by the number of fetuses: the relative abundance of *Prevotella*, *Prevotellaceae_UCG-003*, and *Prevotellaceae_UCG-001* in the PL ewes were higher (*p* < 0.05) than those in the SL ewes, while *unidentified*, *uncultured*, *Ruminococcus*, *Butyrivibrio*, *Christensenellaceae_R-7_group*, *Lachnospiraceae_AC2044_group*, *Lachnospiraceae_XPB1014_group*, and *Anaeroplasma* were lower in the PL ewes (*p* < 0.05) than in the SL ewes.

## 4. Discussion

The imbalance between the increased energy requirements and the available energy in ewes in late gestation can induce metabolic stress, trigger inflammatory responses, and increase the production of ROS [38]. This changes the metabolic state and exposes the ewes to the risk of rumen microbiota imbalance [39], resulting in an increase in metabolic and infectious diseases [40]. The results of this study confirmed that there were differences in the energy metabolism and rumen microbiota of ewes with different numbers of fetuses, and that triplet-fetal ewes had more severe immune responses and oxidative stress.

There are significant differences in feed requirements between the stages of pregnancy [41]. A previous report showed that the DMI of perinatal ewes varied significantly between 1.2 and 2.8 kg/d [19]. Moreover, there are greater nutritional requirements during later gestation, as approximately two-thirds of fetal growth occurs during this period [42]. The uteri of the triplet-fetal ewes in late pregnancy weighed 25% more than those of the twin-fetal ewes [43], suggesting that triplet-fetal ewes face higher metabolic challenges in late gestation to meet the energy requirements of the uterine placenta. It was speculated that the larger uterine volume of triplets resulted in a decrease in the available abdominal space of the rumen, thus affecting the feed intake of the ewes. However, there was no difference in the rumen volume between the single- and multi-fetal ewes, so the effect of the uterus on rumen volume was negligible [43]. This research found no difference in the DMI among ewes carrying different numbers of fetuses, indicating that there was an upper limit of DMI in ewes during late gestation.

The nutritional requirements of ewes in late gestation do not match the feed intake [44], and ewes often rely on fat mobilization to make up for energy deficiencies [45]. The BCS is a subjective measure of the body’s energy reserves [46], and the results of this study found that triplet-fetal ewes were characterized by a lower BCS, possibly because they were more likely to be subjected to nutritional stress than single- or twin-fetal ewes. Triplet-fetal ewes under low BCS management are prone to metabolic diseases and dystocia, with higher mortality [7]. Therefore, producers should develop management guidelines for multi-fetal ewes to maximize their energy levels in late gestation, so as to improve ewes’ ability to cope with metabolic and physiological stress.

Nutrition is a key factor in placental and fetal growth, and fetal growth restriction and high mortality in multi-fetal ewes may be related to insufficient nutrient and metabolite supply [47], which is consistent with the decrease in serum glucose concentration observed with increasing number of fetuses in this study. Maternal glucose is transported across the placenta to the fetus by facilitated diffusion [48]. If maternal hypoglycemia occurs, the flux of glucose into the umbilical cord circulation reduces, and this pattern of maternal–fetal glucose regulation protects the ewe’s brain from glucose shortages during hypoglycemia [21]. Furthermore, it has been found that multi-fetal ewes seem to be more sensitive to hypoglycemic stress due to reduced glucose production and turnover, which makes ewes more susceptible to pregnancy toxemia [49]. When maternal energy is deficient, the mobilization of long-chain fatty acids in adipose tissue increases, and ketone bodies are formed when NEFA production exceeds the oxidative capacity of the liver [50]. Ketone bodies can be used as substitute energy for many tissues, including the placenta. However, the BHBA produced by ewes cannot be utilized by the fetuses, which results in the decreased utilization of BHBA by ewes in late gestation [51]. This study found that the BHBA and NEFA concentrations increased with the increase in the number of fetuses, which may be caused by the increase in BHBA production in the liver of multi-fetal ewes and the decrease in BHBA utilization. A previous study reported that a BHBA concentration of between 0.8 and 1.6 mmol/L indicates that the ewe is suffering from NEB [5]. In this study, the BHBA concentration in triplet-fetal ewes was 1.12 mmol/L 7 days before lambing, which was higher than that of single- and twin-fetal ewes, indicating a higher negative energy balance in the multi-fetal ewes.

Oxidative stress is the pathological basis of cell damage, functional disorders, and various metabolic diseases, while immunosuppression is the result of the combined effects of oxidative stress, endocrine disorders, and other factors [52,53]. Body fat mobilization is an important way for NEB ewes to obtain energy, but it increases the content of BHBA and NEFA in the liver [54], which not only affects the process of lipid metabolism in the body, but also triggers oxidative stress and promotes the production of ROS. We found that MDA, the lipid peroxidation product, was significantly increased and the concentrations of antioxidant factors SOD and GSH-Px were decreased in the late gestation period of the triplet-fetal ewes, suggesting that the protective effect of antioxidant enzymes on lipid peroxidation was reduced. Moreover, with the increase in the BHBA concentration, the degree of lipid peroxidation and the consumption level of GSH-Px in multi-fetal ewes were significantly increased [12]. We found that, regardless of the number of fetuses, the concentration of immune cytokines was significantly higher on day 7 than on day 21 before lambing. This finding is similar to that of Caroprese et al., who found that the concentration of APP was higher at lambing compared with 2 weeks before lambing, and the change in the APP concentration depended on the relative time of delivery and the number of lambs [22]. At the same time, IL-6 is considered a stress indicator related to lambing and an inducer of APP production in the liver [16]. We found that the concentrations of cytokines IL-1β, IL-2, IL-6, and TNF-α were correlated with the number of fetuses, and increased with the increase in the number of fetuses on day 21 before lambing. The secretion of cytokines may be related to a variety of factors. On the one hand, it may be due to the higher concentration of circulating reproductive hormones: the estrogen levels in multi-fetal ewes are significantly higher and promote the secretion of cytokines [55]. On the other hand, it may be related to the oxidative stress caused by the nutritional imbalance in multi-fetal ewes [56]. Immune cells are particularly sensitive to oxidative stress because their cell membranes contain high concentrations of polyunsaturated fatty acids, which are susceptible to peroxidation and produce a large number of ROS under external stimulation. Therefore, as the number of fetuses increases, the ewes suffer a more intense catabolic state to ensure normal fetal growth and development in late gestation, which leads to changes in the antioxidant defense system and inflammatory response.

The gastrointestinal microbiota plays an important role in host energy homeostasis and physiological processes in response to energy deficiency [57]. Chao1 and Shannon indices were used to assess the richness of the microbiota. In this study, no difference was observed in terms of α diversity, and the rumen microbial species were similar in ewes in late gestation with different numbers of fetuses, which may be attributed to them being fed the same diet. Chen et al. found that high-reproductive-capacity sows exhibited higher α diversity at day 30 of gestation, but this difference disappeared at day 110 of gestation [29]; however, Shao et al. reported that the microbial α diversity of high-reproductive-capacity sows in late gestation was lower than that of low-reproductive-capacity sows [58]. These inconsistent results may be due to differences in species and sampling time. However, this study found significant differences in β diversity between the ewes in late gestation with different numbers of fetuses, which is consistent with the results of studies on sows [29,58], suggesting that β diversity may be a key factor in assessing the effects of gastrointestinal microorganisms on the reproductive performance of female animals [59].

Firmicutes and Bacteroidetes were found to be the most abundant bacteria in the rumen in this study. The microbiota of Firmicutes mainly degrades fibrous substances, which is closely related to acetic acid and milk fat production and fat deposition, while Bacteroidetes is the decomposer of non-fibrous plant polysaccharides and proteins in the rumen, and its relative abundance is positively correlated with the production of propionate [60]. Ewes carrying two fetuses are more susceptible to metabolic stress due to higher nutritional requirements [49]. Grain feeding can benefit some microorganisms, such as Bacteroidetes [61,62]. One study monitoring silage intake found that NDF intake decreased with the increase in gestational weeks and fetal numbers [63]. The results of this research showed that the relative abundance of Firmicutes decreased and that of Bacteroides increased in twin- and triplet-fetal ewes compared to single-fetal ewes. The differences in rumen bacteria phyla may be due to the different utilization of dietary ingredients by ewes with different numbers of fetuses, and multi-fetal ewes tend to select grains over fiber to meet their energy requirements.

A previous study found that hepatic cholestasis is correlated with the decline in *Ruminococcus* and *Butyrivibrio* [64]. Meanwhile, these two bacteria are negatively correlated with long-chain fatty acid concentrations [65]. These results showed that the relative abundances of *Ruminococcus* and *Butyrivibrio* of triplet-fetal ewes decreased, which may be related to the increase in serum NEFA concentration, indicating that multi-fetal ewes may experience fat mobilization, liver function impairment, and bile acid secretion disorders. *Christensenellaceae_R-7_group* is a known producer of butyrate [66], which has been identified as an indicator of a healthy digestive system [67]. Christensenellaceae and Lachnospiraceae have been associated with residual feed intake (RFI) in ruminants and are significantly enriched in low-RFI heifers [68]. In this study, we observed that the relative abundances of *Christensenellaceae_R-7_group*, *Lachnospiraceae_AC2044_group*, and *Lachnospiraceae_XPB1014_group* were higher in the rumen of single-fetal ewes. This suggests the more complete fermentation of nutrients and an increase in absorbable nutrients in single-fetal ewes compared to multi-fetal ewes. *Anaeroplasma* has been shown to participate in the tryptophan metabolic pathway, and tryptophan metabolites can upregulate the relative abundance of the beneficial bacteria *Ruminococcus* and *Anaeroplasma* and reduce the inflammatory response of asthmatic mice [69]. In our study, the decrease in the relative abundance of *Anaeroplasma* in the rumen of multi-fetal ewes may trigger the inflammatory response, which is consistent with the elevated concentrations of immune cytokines we observed. *Prevotella* (a member of the Prevotellaceae family) is an anaerobic Gram-negative bacterium of Bacteroides with strong propionate production capacity. The increased relative abundance of *Prevotella* not only participates in glucose metabolism and insulin resistance [70], but also interacts with the immune system and plays a role in the occurrence of inflammatory diseases. *Prevotella* predominantly activates Toll-like receptor 2, leading to the production of Th17-polarizing cytokines by antigen-presenting cells, promoting the mucosal immune response and neutrophil recruitment [71]. We observed that the relative abundance of *Prevotella*, *Prevotellaceae_UCG-003*, and *Prevotellaceae_UCG-001* in the rumen of twin- and triplet-fetal ewes was higher than those of single-fetal ewes. This results in triplet-fetal ewes producing more propionate to participate in the production of glucose to maintain the body’s energy homeostasis, as well as leading to a strong inflammation response.

## 5. Conclusions

In this study, we found that triplet-fetal ewes had higher BHBA concentrations; lower glucose concentrations, BCS, and antioxidant capacities; and were prone to inflammatory responses. The relative abundance of Bacteroidota was higher and that of Firmicutes was lower in the rumen of the triplet-fetal ewes. The reason for the differences in the rumen microbiota in ewes with different numbers of fetuses and the influence on physical metabolism need to be further studied.

## Figures and Tables

**Table 1:** Diet composition and nutrient levels of the basal diet (dry matter basis).

Item	Content (%)
Ingredient	
Silage	38.46
Peanut vine	15.38
Green hay	15.38
Corn	14.38
Bran	3.13
Soybean meal	11.65
CaHPO_4_	0.13
Premix ^1^	0.67
Sodium bicarbonate	0.40
NaCl	0.42
Total	100.00
Nutritional indicator	
Metabolizable energy, ME/(MJ kg^−1^) ^2^	9.40
Crude protein, CP	13.49
Ether extract, EE	2.60
Neutral detergent fiber, NDF	43.16
Acid detergent fiber, ADF	18.91
Calcium, Ca	0.66
Phosphorus, P	0.33

^1^ Provided per kilogram of diet (dry matter): vitamin A 4402 IU, vitamin D 755 IU, vitamin E 126 IU, Cu 12.50 mg, Mn 28.30 mg, Zn 37.74 mg, Fe 40.88 mg, Co 0.85 mg, I 0.97 mg, and Se 0.85 mg. ^2^ ME was calculated based on the metabolizable energy of feed ingredients and their proportions, referring to Sheep and Goat Production (2019)—Feed Composition and Nutritional Value, and other nutritional indicators are the measured values.

**Table 2:** The DMI, BW, and BCS of ewes in late gestation with different numbers of fetuses.

Item ^1^	Time	SL	TL	PL	SEM	*p*-Value ^2^		
						Group	Time	Group × Time
DMI, kg/d	Q21	1.32	1.35	1.33	0.022	0.311	<0.001	0.590
	Q7	1.21	1.23	1.24	0.019			
BW, kg	Q21	55.77	58.23	58.6	1.096	0.604	0.240	0.906
	Q7	58.50	59.7	60.58	1.126			
BCS	Q21	2.79 ^a^	2.74 ^ab^	2.47 ^b^	0.337	0.002	0.001	0.821
	Q7	2.57 ^a^	2.40 ^a^	2.20 ^b^	0.367			

^1^ DMI, dry matter intake; BW, body weight; BCS, body condition score. ^2^ SL, single-fetal ewes; TL, twin-fetal ewes; PL, triplet-fetal ewes. Q21 and Q7, days 21 and 7 before lambing. Values within a row with different superscripts differ significantly at *p* < 0.05.

**Table 3:** Serum energy metabolites of ewes in late gestation with different numbers of fetuses.

Item ^1^	Time	SL	TL	PL	SEM	*p*-Value ^2^		
						Group	Time	Group × Time
BHBA, mmol/L	Q21	0.47 ^b^	0.50 ^b^	0.76 ^a^	0.046	<0.001	0.037	0.276
	Q7	0.47 ^c^	0.67 ^b^	1.12 ^a^	0.093			
NEFA, mmol/L	Q21	0.18 ^b^	0.26 ^b^	0.48 ^a^	0.045	<0.001	<0.001	0.061
	Q7	0.30 ^c^	0.52 ^b^	0.88 ^a^	0.080			
Glu, mmol/L	Q21	2.69 ^a^	1.94 ^b^	1.69 ^c^	0.202	<0.001	<0.001	0.097
	Q7	2.28 ^a^	1.62 ^b^	1.34 ^c^	0.128			
TG, mmol/L	Q21	0.55 ^a^	0.37 ^b^	0.28 ^c^	0.041	0.002	0.327	0.742
	Q7	0.58 ^a^	0.39 ^ab^	0.34 ^b^	0.482			
TC, mmol/L	Q21	2.99 ^a^	2.15 ^b^	1.54 ^c^	0.181	<0.001	0.135	0.822
	Q7	2.76 ^a^	1.90 ^b^	1.51 ^b^	0.184			

^1^ BHBA, β-hydroxybutyrate; NEFA, non-esterified fatty acid; Glu, glucose; TG, triglycerides; TC, total cholesterol. ^2^ SL, single-fetal ewes; TL, twin-fetal ewes; PL, triplet-fetal ewes. Q21 and Q7, days 21 and 7 before lambing. Values within a row with different superscripts differ significantly at *p* < 0.05.

**Table 4:** Serum immune cytokine concentrations of ewes in late gestation with different numbers of fetuses.

Item ^1^	Time	SL	TL	PL	SEM	*p*-Value ^2^		
						Group	Time	Group × Time
IL-1β, ng/L	Q21	12.18 ^c^	16.60 ^b^	19.89 ^a^	1.118	0.327	<0.001	0.553
	Q7	25.31	26.79	25.19	1.899			
IL-2, ng/L	Q21	20.67 ^b^	23.77 ^b^	36.95 ^a^	2.636	<0.001	0.028	0.055
	Q7	26.70 ^b^	30.26 ^ab^	35.64 ^a^	1.028			
IL-6, ng/L	Q21	46.51 ^b^	43.22 ^b^	64.71 ^a^	3.441	0.031	0.007	0.221
	Q7	59.77	66.61	66.52	3.534			
TNF-α, ng/L	Q21	34.16 ^c^	41.8 ^b^	70.83 ^a^	5.542	0.015	<0.001	0.098
	Q7	75.05	82.15	78.29	4.509			

^1^ IL: interleukin; TNF-α: tumor necrosis factor α. ^2^ SL, single-fetal ewes; TL, twin-fetal ewes; PL, triplet-fetal ewes; Q21 and Q7, time (days) before lambing. Values within a row with different superscripts differ significantly at *p* < 0.05.

**Table 5:** Serum antioxidant indices of ewes in late gestation with different numbers of fetuses.

Item ^1^	Time	SL	TL	PL	SEM	*p*-Value ^2^		
						Group	Time	Group × Time
MDA, nmol/mL	Q21	1.03 ^b^	1.32 ^b^	3.36 ^a^	0.374	<0.001	0.001	0.532
	Q7	1.69 ^c^	2.66 ^b^	4.01 ^a^	0.320			
T-AOC, mmol/L	Q21	0.32 ^a^	0.18 ^b^	0.14 ^b^	0.026	0.018	0.143	0.126
	Q7	0.34	0.23	0.20	0.029			
SOD, U/mL	Q21	168.63 ^a^	148.75 ^a^	127.75 ^b^	8.235	<0.001	<0.001	0.550
	Q7	146.94 ^a^	136.98 ^a^	107.75 ^b^	6.545			
GSH-Px, U/mL	Q21	76.86 ^a^	61.44 ^b^	50.20 ^b^	4.815	0.002	0.430	0.054
	Q7	83.66 ^a^	70.03 ^a^	49.42 ^b^	4.923			

^1^ MDA, malondialdehyde; T-AOC, total antioxidant capacity; SOD, superoxide dismutase; GSH-Px, glutathione peroxidase. ^2^ SL, single-fetal ewes; TL, twin-fetal ewes; PL, triplet-fetal ewes. Q21 and Q7, days 21 and 7 before lambing. Values within a row with different superscripts differ significantly at *p* < 0.05.

**Table 6:** Rumen bacteria at the phylum level for ewes in late gestation with different numbers of fetuses.

Item	Time	SL	TL	PL	SEM	*p*-Value ^1^		
						Group	Time	Group × Time
Firmicutes	Q21	49.42 ^a^	36.05 ^b^	33.54 ^b^	2.671	<0.001	0.98	0.194
	Q7	48.04 ^a^	40.06 ^b^	39.96 ^b^	1.687			
Bacteroidota	Q21	44.18 ^b^	57.44 ^a^	60.66 ^a^	2.705	<0.001	0.076	0.233
	Q7	44.91 ^b^	53.14 ^a^	52.40 ^a^	1.964			
Patescibacteria	Q21	2.12 ^a^	2.51 ^a^	2.08 ^a^	0.166	0.101	0.147	0.126
	Q7	3.21 ^a^	2.61 ^ab^	1.98 ^b^	0.224			

^1^ SL, single-fetal ewes; TL, twin-fetal ewes; PL, triplet-fetal ewes. Q21 and Q7, days 21 and 7 before lambing. Values within a row with different superscripts differ significantly at *p* < 0.05.

**Table 7:** Rumen bacteria at the genus level for ewes in late gestation with different numbers of fetuses.

Item	Time	SL	TL	PL	SEM	*p*-Value ^1^		
						Group	Time	Group × Time
*Prevotella*	Q21	15.68 ^b^	23.96 ^a^	27.27 ^a^	1.982	<0.001	0.711	0.891
	Q7	15.95 ^b^	23.85 ^a^	29.06 ^a^	2.210			
*unidentified*	Q21	11.15 ^a^	6.39 ^b^	5.74 ^b^	0.886	<0.001	0.869	0.921
	Q7	11.19 ^a^	6.29 ^b^	5.98 ^b^	0.861			
*uncultured*	Q21	4.13 ^a^	2.76 ^b^	2.66 ^b^	0.282	<0.001	0.600	0. 958
	Q7	4.01 ^a^	2.63 ^b^	2.54 ^b^	0.271			
*Ruminococcus*	Q21	3.94 ^a^	1.77 ^b^	1.52 ^b^	1.159	<0.001	0.261	0. 853
	Q7	3.83 ^a^	1.63 ^b^	1.27 ^b^	0.416			
*Butyrivibrio*	Q21	5.12 ^a^	2.16 ^b^	1.32 ^b^	0.603	<0.001	0.423	0.054
	Q7	3.63 ^a^	2.46 ^ab^	1.72 ^b^	0.350			
*Prevotellaceae_UCG-003*	Q21	1.68 ^b^	3.00 ^a^	3.31 ^a^	0.287	<0.001	0.105	0.918
	Q7	1.42 ^b^	2.59 ^a^	2.83 ^a^	0.251			
*Christensenellaceae_R-7_group*	Q21	3.01 ^a^	1.56 ^b^	1.37 ^b^	0.295	<0.001	0.058	0.636
	Q7	3.90 ^a^	1.81 ^b^	1.94 ^b^	0.387			
*Prevotellaceae_UCG-001*	Q21	1.59 ^b^	3.13 ^a^	3.07 ^a^	0.319	0.005	0.898	0.370
	Q7	1.94 ^b^	2.44 ^ab^	3.29 ^a^	0.251			
*Lachnospiraceae_AC2044_group*	Q21	2.62 ^a^	1.26 ^b^	0.83 ^b^	0.280	<0.001	0.873	0.355
	Q7	2.28 ^a^	1.28 ^b^	1.07 ^b^	0.218			
*Lachnospiraceae_XPB1014_group*	Q21	1.63 ^a^	0.65 ^b^	0.59 ^b^	0.185	<0.001	0.372	0.590
	Q7	1.93 ^a^	0.81 ^b^	0.64 ^b^	0.229			
*Anaeroplasma*	Q21	1.67 ^a^	1.31 ^a^	0.61 ^b^	0.171	<0.001	0.15	0.533
	Q7	1.03 ^a^	1.06 ^a^	0.29 ^b^	0.161			

^1^ SL, single-fetal ewes; TL, twin-fetal ewes; PL, triplet-fetal ewes. Q21 and Q7, days 21 and 7 before lambing. Rumen bacteria genera with relative abundance greater than 1% and significant differences between groups are listed. Values within a row with different superscripts differ significantly at *p* < 0.05.

## Data Availability

The datasets generated in the current study are available in the Genome Sequence Archive repository (http://gsa.big.ac.cn, accessed on 14 March 2024) under accession number CRA015355.
